# Sodium Glucose Co-Transporter Inhibition, an Expanding Impact

**DOI:** 10.31083/j.rcm2405130

**Published:** 2023-04-26

**Authors:** Badr Harfouch, Robert Chilton

**Affiliations:** ^1^Sinai Cardiology Faculty Group, LifeBridge Health, Baltimore, MD 21209, USA; ^2^Division of Cardiology, Department of Medicine, University of Texas Health Science Centre at San Antonio, San Antonio, TX 78229, USA

The importance of sodium glucose co-transporter type II inhibitors (SGLT2i) has been significantly increasing considering the positive outcome reported in several studies, among heart failure patients either with preserved or reduced ejection fraction and even in the absence of diabetes.

Glucose is normally filtered in the kidney and is reabsorbed in the proximal tubules. The sodium-glucose co-transporter 2 (SGLT2) is responsible for transporting 98% of urinary glucose across the proximal tubule membrane. When the SLC5A2 gene mutates, it leads to a defective SGLT2 protein, resulting in the significant glycosuria, with modestly decrease in blood pressure and weight. On the other hand, the defective SGLT2 was not associated with significant adverse effects related to the glycosuria [1]. This special edition included reviews the role of SGLT2 inhibitors on many different aspects.

In patients with established atherosclerotic cardiovascular disease, SGLT2i provide clinical benefits by reducing the risk of myocardial infarction, stroke, or cardiovascular death. However, patients with many risk factors do not have the same benefits. On the other hand, SGLT2i significantly reduces the risk of hospitalization due to heart failure or progression of renal disease, regardless of the presence of atherosclerotic cardiovascular disease or heart failure at baseline [2]. While the exact beneficial mechanism of SGLT2i on the cardiovascular system is still not fully clear, Aguiar-Nevis at all, reviewed the available data to clarify the cardioprotective mechanism behind SGLT2i. In patients with heart failure preserved ejection fraction, SGLT2i were the only class of drug that have been proven to change cardiovascular outcomes in a consistent and transversal manner, independent of age, functional class, or diabetes status. The cardiovascular and renal benefits are due to multifactorial mechanism and cannot be explained only by SGLT2i effect on glycemic control [3].

Several reviews have shown the metabolic effects of SGLT2i. Compared to a placebo, all SGLT2 inhibitors were found to enhance glucose control by reducing HbA1c levels by 0.6% to 0.9% and fasting plasma glucose (FPG) levels by 1.1 mmol/L to 1.9 mmol/L, as well as reduce body weight by 1.6 kg to 2.5 kg, systolic blood pressure by 2.8 mmHg to 4.9 mmHg, and diastolic blood pressure by 1.5 mm Hg to 2.0 mmHg. Furthermore, all SGLT2 inhibitors showed a minor increase in high density lipid (HDL)-cholesterol levels compared to the placebo (the highest increase being 0.07 mmol/L). According to the available evidence, SGLT2 inhibitors were found to result in a slight increase in low density lipid (LDL)-cholesterol levels and a reduction in triglycerides with both doses of canagliflozin when compared to a placebo. However, due to limited data on total cholesterol, further investigation is needed to understand the effects of SGLT2 inhibitors on the overall lipid profile. The changes in these cardiometabolic biomarkers indicate a theoretical potential for microvascular and cardiovascular benefit [4]. Sanz-Cánovas *et al*. [5] examined the latest evidence on the impact of SGLT2i on various metabolic disorders, including hypertension, dyslipidemia, obesity, vascular aging, osteoporosis, and nonalcoholic fatty liver disease. SGLT2i showed benefits in non diabetic patients with obesity, hypertension and hyperuricemia. However, other metabolic disorders require further studies [5].

The promising role of SGLT2i in patients with acute coronary syndromes is being investigated. A potential key benefit of SGLT2 inhibition is related to the attenuation of neurohormonal activation, cardiomyocyte necrosis, and reperfusion injury. Furthermore, it is believed that SGLT2 inhibition can enhance outcomes by improving endothelial function and vasodilatation, boosting myocardial energy metabolism, preserving cardiac contractility, and reducing pathways of oxidative stress. This eventuality can lead to an improvement in coronary blood flow and ventricular unloading. These effects may further prevent cardiomegaly, dysrhythmia, fibrosis, and cardiac failure. In the post-myocardial infarction (MI) population who has a high risk of cardiometabolic disease, SGLT2 inhibition may offer additional benefits such as decreasing afterload and preload, improving glycemic control, and promoting weight loss through natriuresis and glucosuria [6]. Benedikt *et al*. [7] presented the evolving trials assessing important knowledge as to the beneficial benefit of early initiation of SGLT2 inhibitors after acute massive myocardial infarction. At least in one randomized controlled trial, SGLT2i group showed better outcome surrogated by N-Terminal pro Brain Natriuretic Peptide (NT-proBNP), cardiac chamber sizes and functional parameters, as well as placebo like safety profile [7].

All members of society need to be represented in medical research, but there is major under-representation of racial and ethnic minorities in clinical trials. There was a tendency for non-white participants to be underrepresented in industry-funded trials, even though they bear a significant disease burden. Like several other trials, the SGLT2i study (such as the EMPA-REG results) represented an outlier in this trend, with a nonwhite participation-to-prevalence ratio of 1.18 [8]. Nasser *et al*. [9] assessed a sensitive and extremely important topic regarding the differences in racial and ethnic representation of the currently available evidence related to SGLT2i. Despite the under representation of minorities such as non hispoanic black population, SGLT2i showed benefits on this population patients who have heart failure (reduced or preserved), overall blood pressure lowering effect, chronic kidney disease and weight loss, even in the absence of diabetes [9].
